# Recycling Technologies for Biopolymers: Current Challenges and Future Directions

**DOI:** 10.3390/polym16192770

**Published:** 2024-09-30

**Authors:** Adenike A. Akinsemolu, Adetola M. Idowu, Helen N. Onyeaka

**Affiliations:** 1 School of Chemical Engineering, University of Birmingham, Birmingham B15 2TT, UK; 2 The Green Institute, Ondo 351101, Nigeria; 3 Faculty of Life Sciences, Rhein-Waal University of Applied Science, 47533 Kleve, Germany; micheal.idowu@rocketmail.com

**Keywords:** biopolymer, sustainability, recycling, biodegradable, application

## Abstract

Plastic pollution is a major driver of climate change that is associated with biodiversity loss, greenhouse gas emissions, and negative soil, plant, animal, and human health. One of the solutions that has been proposed and is currently reducing the adverse effects of plastic on the planet is the replacement of synthetic plastics with biopolymers. The biodegradable polymers have been adapted for most of the applications of synthetic plastic. However, their use and disposal present some sustainability challenges. Recycling emerges as an effective way of promoting the sustainability of biopolymer use. In this article, we review recycling as a viable solution to improve the sustainability of biopolymers, emphasizing the current types and technologies employed in biopolymer recycling and the challenges faced in their adoption. Our exploration of the future directions in the conversion of biopolymers into new polymers for reuse establishes a connection between established continuous technological innovation, integration into circular economy models, and the establishment and strengthening of collaborations among key stakeholders in relevant industries as necessary steps for the adoption, full utilization, and improvement of recycling technologies for biopolymers. By connecting these factors, this study lays a foundation for the establishment of a roadmap for improved biopolymer recycling technologies and processes that promote the sustainability of synthetic plastic alternatives.

## 1. Introduction

Planet Earth is facing an existential crisis brought about by climate change and occasioned by irreversible fluctuations in weather patterns, rapid biodiversity loss, an increase in the incidence of food, water, and vector-borne diseases and pathogenic infections, and an increase in the incidence of natural disasters [1]. One of the primary factors driving climate change is the continued reliance on fossil fuels, both as a source of energy and as a raw material for products such as plastic [2]. Since their large-scale manufacture began in the 1950s, the production and use of plastics have become widespread, owing to the material’s diverse applications across industries [3]. Plastic is produced from monomers, the majority of which are produced from fossil hydrocarbons [3]. Consequently, most of the plastics used across the world are synthetic and non-biodegradable. The non-biodegradable nature of plastics made from fossil fuels is one of the major sources of pollution across all ecosystems. Currently, plastic makes up an estimated 5–12% of the world’s waste by composition and between 20–30% of global waste by weight [4]. The waste is associated with adverse environmental impacts such as clogged water and drainage systems, contaminated water, impaired soul structure and fertility, and equally adverse impacts on human and animal health, including metabolic disorders, neurotoxicity, oxidative stress, and general organ dysfunction [4,5]. Despite their evidently harmful impacts on the environment and the health of humans, as well as aquatic and terrestrial animals, the production, use, and disposal of plastics are projected to keep growing in the foreseeable future, with the volume of plastic waste expected to double by 2030 and triple or quadruple by 2050 [6].

The projected growth in plastic production, use, and disposal necessitated the development of innovative solutions to mitigate the extensive damage they cause to the environment and the overall health of the planet’s life. One of the innovative solutions to this end is biopolymers. Biopolymers are biodegradable polymers that have been fronted as viable alternatives to synthetic plastic [7]. Biopolymers are made from organic substances that are derived from natural sources such as nucleic acids, lipids, proteins, and carbohydrates, often relying on microorganisms for synthesis [8]. Biopolymers are increasingly used to replace plastics in industries such as agriculture, pharmaceuticals, food packaging, cosmetics, and biomedicine [9]. Their adoption helps reduce emissions from fossil fuel extraction and plastic production, lowers the risk of microplastic contamination in humans and animals, and decreases pollution and toxicity associated with synthetic plastic disposal.

Whilst effective and highly viable as replacements for synthetic plastics, replacing synthetic plastics with bioplastics made from biopolymers presents problems of its own as well. First, they are produced from natural sources that have other uses. For instance, some polysaccharides used in the production of biopolymers are produced from feedstocks such as maize [8]. This repurposing of land from the production of food for consumption to the production of materials for bioplastic production causes competition for resources between bioplastics and the animals and human beings who use the crops as a source of food [10]. Second, while biodegradable, bioplastics at the end of their life cycle still accumulate in various ecosystems awaiting the decomposition process, creating pollution [11]. Further, the decomposition of bioplastics in conditions such as composting produces methane, contributing to greenhouse gas emissions [10]. Third, bioplastics are relatively more expensive to produce compared to conventional plastics [12]. All three problems can be solved through recycling, which will reduce the amount of plastic waste, both synthetic and biodegradable, that ends up in the environment, promote the cost-effectiveness of bioplastic use, and reduce the rate of the repurposing of land to produce feedstocks for the production of bioplastics. Consequently, there is a growing need for effective recycling technologies for bioplastics to enhance their viability as better alternatives to synthetic plastics. To this end, we explore some of the recycling technologies for bioplastics, the challenges that hinder or could affect their adoption, and future directions in the recycling of bioplastics. The primary purpose of this study is to synthesize the existing knowledge on biopolymer recycling, identify ways to improve recycling technologies, emphasize the need for continuous innovation, and propose collaborative pathways to achieve these goals. This study can serve as a foundation for further exploration of strategies to adapt both existing and new recycling technologies while leveraging partnerships and expertise across sectors to enhance biopolymer recycling and promote the sustainability of plastic alternatives.

## 2. Overview of Biopolymers

### 2.1. Definition and Types of Biopolymers

The origin, history, composition, and properties of biopolymers have been researched extensively and documented in the literature, either independently or as part of preludes to their application across various industries. Different accounts of their origin are documented but a consensus emerges of their discovery in the 19th century. The first account attributes the identification of biopolymers to the discovery of collagen in 1938, which laid the foundation for its later applications in biopolymers, drug delivery systems, hydrogels, and nanoparticles [13]. A second account traces the origin of biopolymers to a wood-based material produced in the 1850s by mixing sawdust, vegetable oils, and mineral or metallic fillers [14]. A third account links the development of biopolymers to the discovery and characterization of chitosan’s properties over several decades in the early and mid-19th century, which eventually led to the creation of chitosan-based biopolymers [15]. Despite the varied attributions for the discovery of biopolymers, their contributions to the development, characterization, and understanding of biopolymers are significant. Various definitions of biopolymers have been advanced. They all share universal elements, including the description of the structure of the polymers, the nature of the materials used in their production, and how they are synthesized and produced [8,16,17,18]. From a compilation of the different definitions advanced, a biopolymer is a functional biodegradable material produced from organic substances derived from natural sources. The structure of biopolymers comprises chain-like molecules that are made from monomeric units linked together by covalent bonds [9].

**Figure 1:**
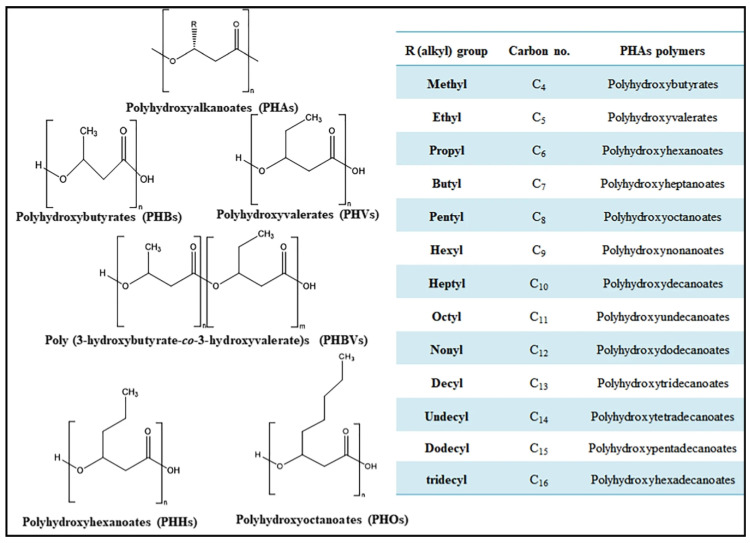
PHA-Based Bioplastics [25].

One of the primary bases of the categorization of biopolymers is their method of production, which yields three categories, namely microorganism-derived biopolymers, vegetal or animal-based biopolymers, and biotechnology-derived biopolymers [19]. Microbial biopolymers are produced by microorganisms, particularly bacteria, through the conversion of carbon and nitrogen into various intracellular or extracellular biopolymers [20]. The defining characteristics of this category of biopolymers include biocompatibility, biodegradability, and ease of modification since they are made up of various functional groups [20]. The most common biopolymers produced through this method are polyhydroxyalkanoates (PHA), a type of biopolyester [19,20,21]. Some PHA-based bioplastics are shown in Figure 1. Other common microbial biopolymers include other polyesters such as polyhydroxybutyrate (PHB) and a variety of polyamides, polysaccharides, and polyphosphates. Comparatively, animal or vegetal biomass produces biopolymers such as chitosan, cellulose, pectin, collagen, whey, gelatin, casein, and hyaluronic acid [22]. Chitosan is primarily produced from the shells of insects and crustaceans, particularly shrimp. Gelatin is produced from raw collagen harvested from bones, skin, and animal connective tissue while cellulose, which is contained in plant cell walls, is produced from plant biomass such as feedstocks. Casein and whey are both produced from dairy milk while hyaluronic acid, a polysaccharide, can be produced from either animal tissue or plants through fermentation. The polymers produced from plants and animals are fully biodegradable, renewable, and biocompatible, making them non-toxic to the cells of any living thing [23]. The third category of biopolymers, which are produced using biotechnological processes, includes polylactic acid (PLA), which is produced through the fermentation of lactic acid from natural sources such as starch [24]. Together, PLA, PHA, and the biopolymers produced from plant and animal biomass are used in the production of biodegradable plastics that have been adapted for the applications for which synthetic plastics are used currently and previously. The various categories of biopolymers are shown in Table 1.

### 2.2. Applications of Bipolymers

Biopolymers have been adapted for various applications across industries that previously relied on plastic as raw materials for consumer products, packaging material, and as components of machinery used in the production of the industries’ products. These applications have been studied and documented extensively in the literature. From the literature, some of the industries in which biopolymers are used include the agriculture, pharmaceuticals, bioengineering, water treatment, biomedical, packaging, cosmetics, food, construction, and pulp and paper industries [9,26,27]. The specific applications of biopolymers in these industries mirror the applications of synthetic plastics in these sectors. In the biomedical and pharmaceutical industries, for instance, biopolymers have been adapted for drug delivery systems, wound healing materials, and tissue engineering according to various studies that explore the general applications of the polymers [9,26,28]. Other studies delve deeper into the applications of biopolymers in the two industries, explaining the mechanisms that render the biopolymers viable for the highlighted applications and the biotechnological technologies used in the formulation of the biodegradable polymers. Notably, Opris et al. (2024) [29] explore the applications of biopolymers such as alginate, chitosan, collagen, gelatin, and albumin in drug delivery, Gardikiotis et al. (2022) [30] explore the applications of biopolymers such as alginate, chitosan, hyaluronic acid, and collagen to promote wound healing, and Mahmud, Islam, and Mobarak (2023) [31] explore the mechanisms of chitosan, alginate, and other natural hydrogel polymers that are exploited to make them suitable for tissue regeneration and cell development. A similar trend in the exploration of the applications of biopolymers in other industries was observed, with a combination of general and specific explorations of the uses of polymers well-documented in the literature. The applications highlighted in the food and food packaging industries include the maintenance of food freshness and quality by leveraging their physical, chemical, and biological properties to protect food from contaminants and environmental elements, the detection of food spoilage to promote food safety, and in the production of food as edible films and additives [32,33]. For these applications in packaging, biopolymers based on proteins and polysaccharides such as starch are favored for their advantages, which include cost-effectiveness, ease of availability since the source materials are available in abundance, compatibility with food and drugs, which eliminates the risk of toxicity, and biodegradability, which makes the resulting packaging eco-friendly [7]. However, biopolymers have some drawbacks, particularly their mechanical weakness and high sensitivity to moisture. Fortunately, these challenges are addressed easily through thermal and mechanical modification using other biopolymers such as gelatin [7]. The biopolymers are often reinforced with nanoparticles, which enhance the properties that make the bioplastics suitable for packaging by enhancing their moisture barrier and releasing antimicrobials to prevent contamination while enhancing the recyclability of biopolymers by enhancing their mechanical, thermal, and chemical stability [7,13]. In addition to nanoparticles, biopolymers can be blended together with other compounds such as phenolic compounds, pigments, and essential oils, which enhance beneficial properties such as attractiveness and extended food shelf-life while enhancing the recyclability of the packaging by reducing their brittleness and the rate of moisture absorption [32]. In agriculture, applications of animal- and vegetal-based biopolymers such as chitosan, lignin, alginate, starch, and gelatin, including the delivery of agricultural chemicals, the production of mulch to promote the soil’s ability to retain water, and as a coating for seeds, have been established [34,35]. Ultimately, the applications of biopolymers are well-established in the literature. Table 2 below summarizes some of these applications. However, the links between their growing usage and overall acceptability as viable alternatives to plastics, the implications on environmental pollution, and the need for recycling to enhance their overall sustainability are relatively underexplored by these studies.

## 3. Current Recycling Technologies for Biopolymers

**Figure 2:**
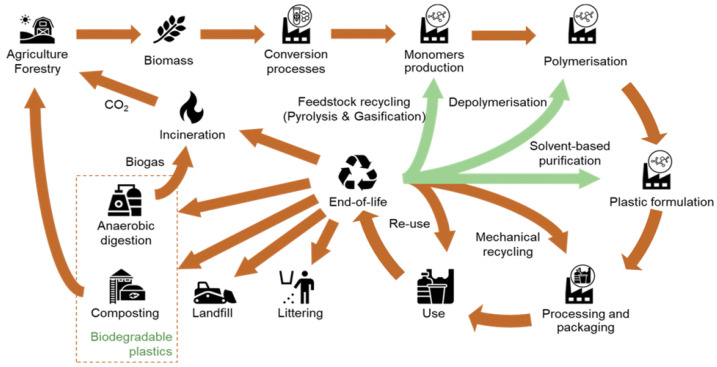
A Depiction of the Sustainability of Bioplastics based on their Life cycle [37].

Whilst most studies that identify the applications of biopolymers or explore them in depth do not link the materials to environmental pollution or establish the need for recycling to make them sustainable, a significant pool of the literature is dedicated to the adverse environmental impacts of biopolymers. The most cited impacts include their implication on food production since they necessitate the use of land previously used for agriculture for the production of biomass, the production of greenhouse gases from their decomposition, and emissions from the chemical processing and synthesis of some biopolymers [10,36]. While the link is not well-established by these studies, the adverse impacts of biopolymer production, use, and disposal warrant the evaluation of the current recycling technologies to promote the overall sustainability of biopolymers, as presented in Figure 2 below.

Various studies on the mechanical recycling process adapted for biopolymers fail to establish the need for recycling but explore the mechanisms, technological advancements, implementation challenges, and benefits of the process. From the available literature, the mechanical recycling of bio-based materials such as biopolymers is a simple five-step process in which used biopolymers are collected and sorted, ground into smaller pieces, separated based on the different properties of different polymers, cleaned, and melted into bioplastic pellets for reuse [38]. The primary benefit of the process, which is the improved sustainability of bioplastics, is highly implied. The limitations, on the other hand, are discussed explicitly, particularly the degradation of biopolymers after mechanical recycling, yielding bioplastics with reduced thermal stability, lower molecular weights, and impaired viscosity [38,39,40]. However, some approaches to improve the properties and quality of recycled biopolymers are highlighted, albeit in passing, creating a gap in the literature on the restoration of the quality of recycled biopolymers. A notable finding on the viability of mechanical recycling in promoting the sustainability of biopolymers is the simplicity of the method, its low cost, and its simple infrastructure [41].

**Figure 3:**
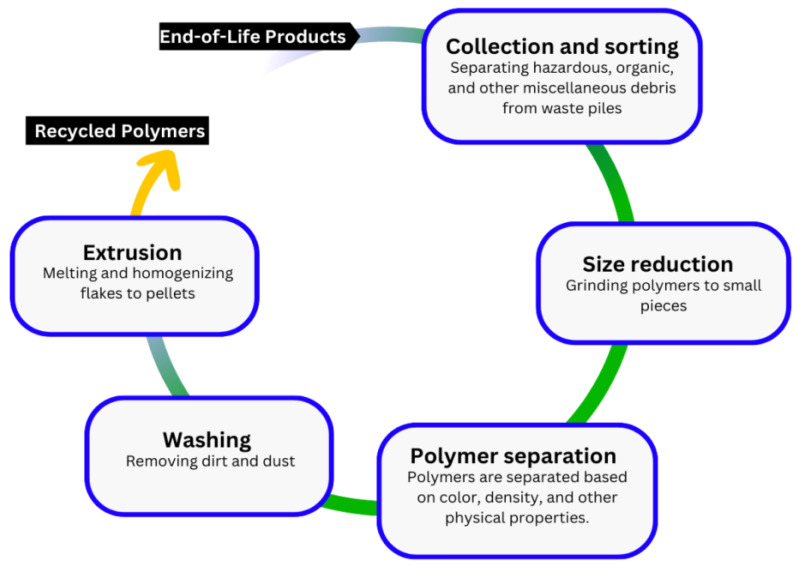
The Chemical Recycling Process for Thermal Biopolymers [38].

Comparatively, chemical recycling has received similar levels of attention, with studies focusing on the process, benefits, and outcomes of the process. The typical chemical recycling process is described as breaking down biopolymers into monomers or smaller molecules, which allows for their regeneration into the same polymers or new products [39]. An example of this process is depicted in Figure 3 below. Unlike mechanical recycling, which follows the same process every time, the process of chemical recycling differs based on the method of recycling adopted. The methods of chemical recycling include the thermal degradation of the polymers, partial or complete hydrolysis, dry heat depolymerization, glycolysis, and alcoholysis, with the final output of each process depending on the method selected and the type of biopolymer to which it is applied [39]. Thermal degradation or pyrolysis uses heat in a chemically inactive environment to break down polymers into biochars, organic vapors, and gasses [39,40]. The organic char is used in the reconstruction of polymers. Hydrolysis, on the other hand, breaks down polymer chains using water as a reactant to yield monomers that can be synthesized again into polymers [40,42]. Glycolysis, a third approach to chemical recycling, involves inserting a glycol into the chain that binds polymers to break the links and replace them with hydroxyl terminals [40]. Each of these methods can be applied to biopolymers but some are limited in applicability to individual polymers at a time while others can be used on mixed waste streams. Pyrolysis, for instance, can be used in the recovery and recycling of polymers from mixed plastic waste while depolymerization lacks the capacity [43,44]. These distinctions, and the suitability of different chemical recycling methods for different biopolymers, call for more research to broaden knowledge and the literature pool on the method of recycling.

**Figure 4:**
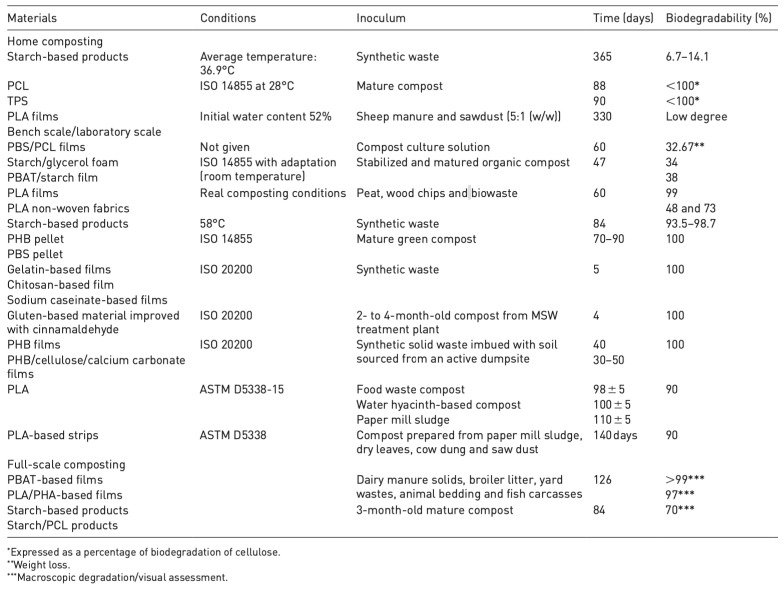
The Biodegradability of Biopolymers under Aerobic Conditions [39].

Finally, the biodegradable property of biopolymers enables the use of biological recycling approaches to break them down. Referred to as organic recycling in some studies, biological recycling leverages natural processes such as composting and enzymatic degradation, with microorganisms facilitating both processes [39]. The approaches to biological recycling break down polymers into biomass, water, and either biogas or carbon dioxide based on the conditions under which the degradation occurs. Notably, the processes for the decomposition of biopolymers differ based on the materials used for their composition, the desired level of decomposition, and the conditions under which the process takes place, warranting further research and the broadening of the existing pool of knowledge on the method of recycling. Nevertheless, Figure 4 below shows the different rates of biodegradability of biopolymers by material, conditions for organic recycling, and the inoculants used in the process. Ultimately, the three recycling methods are indispensable in the pursuit of sustainability since, as per predictions, 2.62 MT of biopolymers were produced globally in 2023 [40].

## 4. Challenges in Recycling Biopolymers

### 4.1. Technical Challenges

Recycling biopolymers presents several technical challenges that arise from the natural properties of these materials. One major issue that has been observed is the variability in the composition of biopolymers. Unlike the conventional plastics that we all know of, biopolymers can be derived from a wide range of biological sources, which then leads to differences in their chemical and physical properties. This variability complicates the recycling process as each type of biopolymer may require different recycling techniques [45].

One of the major challenges in mechanical recycling is the degradation of polymer quality after each cycle. For instance, PHA biopolymers, when recycled, tend to lose molecular weight and mechanical properties such as tensile strength, which limits their applications. Techniques such as blending with virgin materials or enhancing the recycling process through additives are currently being explored to address this issue [37,46].

Another very important technical challenge is the issue of contamination. Biopolymers often come into contact with food and other organic materials, leading to contamination that can hinder or alter the recycling process. Contaminants can interfere with the reprocessing of biopolymers, reducing the quality of the recycled material and making it less suitable for high-value applications [46].

Furthermore, its degradation ability during recycling is also a critical issue. Biopolymers are designed to break down more easily than conventional plastics, which is beneficial for composting but on the contrary, it is very problematic for recycling. The recycling process, particularly mechanical recycling, can cause further degradation of the polymer chains, resulting in a loss of mechanical properties and limiting the range of applications for the recycled material [47].

### 4.2. Economic Challenges

The economic functionality of biopolymer recycling is another major challenge. The costs associated with collecting, sorting, and processing biopolymers can be high. Unlike conventional plastics, biopolymers are not yet produced at the same scale, which results in higher costs for recycling infrastructure and operations [48].

Market demand for recycled biopolymers is also a major economic concern. This is because recycled products often have inferior properties when compared to virgin or new materials, making them less attractive to manufacturers. Additionally, the price of virgin biopolymers has been decreasing, further diminishing the economic incentive to invest in recycling technologies [49].

In addition, infrastructural facilities for biopolymer recycling are not as developed as those for conventional plastics. Many existing recycling facilities are not equipped to handle the specific requirements of biopolymer recycling, leading to higher initial investment costs for upgrading or building new facilities [50].

### 4.3. Regulatory and Policy Challenges

Regulatory and policy barriers significantly affect the development and implementation of biopolymer recycling technologies. The regulations governing waste management and recycling vary widely between different regions and countries, creating a fragmented landscape that complicates the establishment of a standardized recycling process for biopolymers [51]. There is also a lack of clear and consistent definitions and classifications for biopolymers within regulatory frameworks. This ambiguity can lead to uncertainties in compliance and enforcement, discouraging investment in biopolymer recycling technologies [47].

Furthermore, policy support for biopolymer recycling is often insufficient. While there are policies promoting the use of biopolymers, there is a need for more targeted support for their recycling. Incentives such as subsidies, tax breaks, and grants for recycling infrastructure and research can help address some of the economic challenges, but these are currently limited [46].

### 4.4. Chemical Breakdown Pathways

Biopolymer recycling involves breaking down polymer chains into their monomeric components through processes such as hydrolysis and enzymatic degradation. This section explores the chemical breakdown pathways for various biopolymers, emphasizing the challenges and technologies involved.

#### 4.4.1. Molecular-Level Recycling of Polyhydroxyalkanoates (PHAs)

Polyhydroxyalkanoates (PHAs), produced by bacterial fermentation, undergo hydrolysis during chemical recycling. Water molecules react with the polymer, breaking the ester bonds and forming hydroxy acid monomers:

PHA + H_2_O → Hydroxy acid monomers.

Enzymatic degradation can enhance this process, with enzymes such as PHA depolymerase catalyzing the breakdown of PHAs into monomers more efficiently. These monomers can then be repolymerized into new PHAs, maintaining material quality across multiple recycling cycles [52].

#### 4.4.2. Chemical Depolymerization of Polylactic Acid (PLA)

Polylactic Acid (PLA) recycling involves depolymerization, achieved through either thermal degradation or hydrolysis. Water molecules break the ester bonds in the polymer, resulting in lactic acid monomers:

PLA + H_2_O → Lactic acid.

These lactic acid monomers can be repolymerized into new PLA, allowing for high-purity recycling. Enzymatic degradation is another pathway, where specific enzymes catalyze the breakdown of PLA into lactic acid, enabling high-purity reuse [53].

#### 4.4.3. Enzymatic Degradation of Polyethylene Terephthalate (PET)

In PET recycling, enzymatic degradation targets the ester bonds in the polymer. Enzymes such as PETase break down PET into terephthalic acid and ethylene glycol:

PET → Terephthalic acid + Ethylene glycol.

This method offers high-purity recycling, even in mixed or contaminated waste streams, making it a promising technology for PET recycling [53].

## 5. Case Studies and Success Stories

In recycling biopolymers, real-world examples and successful projects offer useful insights into how these technologies can be practically applied. This section discusses some examples of successful biopolymer recycling initiatives, highlighting their approaches, outcomes, and contributions to sustainability. These case studies and success stories not only demonstrate the possibilities of different recycling methods but also inspire further development and innovation in the field.

### 5.1. Case Studies

#### 5.1.1. NatureWorks LLC and PLA Recycling

NatureWorks LLC, a leading producer of PLA biopolymers, operates a large-scale facility capable of processing 150,000 tons of PLA annually. The company’s initiative to enhance PLA recycling through mechanical and chemical processes demonstrates the scalability of biopolymer recycling technologies. In Europe, the Total Corbion PLA plant has a production capacity of 75,000 tons per year, which represents a significant advancement in the recycling infrastructure for PLA biopolymers [54,55].

One notable success in biopolymer recycling is the industrial recycling of polylactic acid (PLA), a widely used biopolymer. PLA is derived from renewable resources such as cornstarch and sugarcane. NatureWorks LLC, a US-based international organization that is a pioneer in the production of polylactic acid (PLA) biopolymers, has made significant strides in the recycling of PLA. NatureWorks’ Ingeo PLA is widely used in packaging, disposable cutlery, and other applications. Recognizing the importance of closing the loop, NatureWorks has invested in technologies and partnerships to enhance the recyclability of PLA products.

One of the key initiatives by NatureWorks is their collaboration with various recycling facilities to improve the mechanical recycling of PLA. By partnering with companies such as Plarco and Synbra, NatureWorks has developed processes that effectively sort and recycle PLA from mixed plastic waste streams. These efforts have led to the successful recycling of PLA into new products, demonstrating the potential for a circular economy in the biopolymer industry [56,57].

A study on the life cycle assessment of PLA conducted by [31] demonstrates the environmental benefits of recycling PLA compared to incineration or landfill disposal. The process involves mechanical recycling where PLA products are collected, cleaned, shredded, and reprocessed into new PLA products. This method not only reduces the demand for virgin materials but also significantly lowers greenhouse gas emissions [58].

#### 5.1.2. PHA Applications in the Medical and Agricultural Sectors

Polyhydroxyalkanoates (PHAs) are a family of biopolymers that are gaining attention for their biodegradability and recyclability. Unlike other bioplastics, PHAs can be broken down by microorganisms in natural environments, making them ideal for recycling through biological methods. However, mechanical and chemical recycling methods have also shown promise in enhancing the recyclability of PHAs.

In the context of mechanical recycling, PHAs are collected, sorted, cleaned, and reprocessed into new products. This process is beneficial for maintaining the physical properties of PHAs, allowing them to be reused in various applications. However, mechanical recycling of PHAs can result in reduced material quality after multiple cycles due to polymer degradation [38].

Chemical recycling offers a more advanced approach by breaking down PHAs into their monomeric components, which can be reused to create new PHA polymers. Methods such as enzymatic degradation have been explored, where specific enzymes are used to break down PHAs into monomers. This method allows for high-purity recycling, making it a promising solution for industries that require consistent material properties, such as the medical and agricultural sectors [53].

In medical applications, recycled PHAs have been successfully used to create biodegradable sutures, implants, and drug delivery systems. These materials can safely degrade in the body, reducing the need for surgical removal and minimizing environmental waste [56].

However, in the agricultural sector, PHAs are recycled to produce biodegradable films and packaging materials. These materials are used to reduce plastic waste in farming practices, such as in biodegradable mulch films that control weeds and retain soil moisture. The recycling of PHAs in agriculture not only contributes to environmental sustainability but also enhances soil health, as these films eventually break down into non-toxic residues that enrich the soil [24].

#### 5.1.3. Recycling of Starch-Based Polymers in Packaging

Starch-based polymers are another category of biopolymers that have found success in recycling initiatives, particularly in the packaging industry. Companies such as Novamont, an Italian-based organization, have developed compostable packaging materials derived from starch that can be recycled through industrial composting processes. Novamont has developed a range of biodegradable and compostable bioplastics that are derived from renewable resources. These products are designed to integrate seamlessly into existing organic waste streams. The company has partnered with waste management facilities to improve composting systems, ensuring that their Mater-Bi products, which are biodegradable and compostable bioplastics, are properly composted at the end of their use. This collaboration has resulted in increased composting rates and reduced contamination in organic waste streams [59].

Novamont’s Mater-Bi products are used in various applications, including shopping bags, food packaging, and agricultural films. In Italy, these Mater-Bi bags are used for organic waste collection. These bags are compostable and have facilitated the efficient collection and recycling of organic waste, reducing the environmental impact of plastic waste. The success of this initiative has led to increased consumer awareness and demand for compostable packaging solutions, driving further innovation and development in the field of starch-based biopolymers [56].

A case study that exemplifies this process is the work done by Carbios, a French company specializing in enzymatic recycling technologies. They developed a process using PETase, an enzyme capable of depolymerizing PET and PLA into their monomers [53]. This enzymatic recycling technology offers a promising method for handling contaminated or degraded biopolymers, contributing to a circular economy model in biopolymer recycling.

#### 5.1.4. Chemical Recycling of Biopolymers

Chemical recycling, involving the breakdown of polymers into their monomers, has shown promise in recycling mixed and contaminated biopolymer waste. A leading example of this is the work done by Carbios, a French company specializing in enzymatic recycling technologies. Carbios has developed a process that uses enzymes to depolymerize PET (polyethylene terephthalate) and PLA back into their monomers, which can then be repolymerized into new plastics.

The success of Carbios’ enzymatic recycling technology lies in its ability to handle a wide range of PET and PLA products, including those that are heavily contaminated or degraded. This technology not only offers a solution to the recycling of mixed plastic waste but also reduces the environmental footprint of plastic production by enabling the reuse of existing materials [56].

#### 5.1.5. Biological Recycling through Composting and Enzymatic Degradation

Biological recycling methods, such as composting and enzymatic degradation, leverage natural processes to recycle biopolymers. One successful example is the use of enzymatic degradation to recycle PHAs. Researchers have developed enzymes that can break down PHAs into their constituent monomers, which can then be used to produce new biopolymers or other value-added products [56]. This method offers a sustainable and environmentally friendly alternative to traditional recycling techniques. The success of this approach has been demonstrated in pilot projects, showing high recovery rates and purity of the recycled materials [59].

Composting is another effective biological recycling method, particularly for biodegradable biopolymers such as PLA and starch-based plastics. Industrial composting facilities can process these materials along with organic waste, converting them into compost that enriches the soil. This approach not only diverts biopolymer waste from landfills but also supports sustainable agriculture by producing high-quality compost [59].

#### 5.1.6. Public-Private Partnerships in Biopolymer Recycling

Public-private partnerships have also played a crucial role in advancing biopolymer recycling. For instance, the collaboration between Imperial College London and the University of California, Davis has led to the development of a systems-thinking framework for biodegradable bioplastic packaging. This partnership has focused on consumer behavior, waste management infrastructure, and policy interventions to enhance the recycling of bioplastics. The project has successfully mapped out consumer behaviour chains and identified key factors influencing the disposal of bioplastics, leading to more effective recycling strategies [59].

### 5.2. Success Stories: Global and Local Initiatives

#### 5.2.1. Germany’s BioCycle Initiative

Germany has been at the forefront of biopolymer recycling through its BioCycle initiative, which promotes the use of biodegradable plastics in packaging and agriculture. This program has successfully integrated biodegradable plastics into the country’s waste management system, resulting in high recycling rates and reduced environmental impact [56].

#### 5.2.2. Japan’s Biomass Town Concept

Japan’s Biomass Town concept involves the establishment of local communities that utilize biopolymers and biomass resources for sustainable development. These communities implement comprehensive recycling programs that include the collection and composting of biodegradable plastics, contributing to a circular economy and reducing reliance on fossil fuels [56].

#### 5.2.3. US-Based Closed Loop Partners

In the United States, Closed Loop Partners has invested in companies that are developing innovative recycling technologies for biopolymers. Their support for projects such as PureCycle Technologies, which focuses on the recycling of polypropylene, demonstrates the potential for public–private partnerships in advancing biopolymer recycling [57].

##### Brazil’s Sugarcane-Based Plastics

Brazil has leveraged its abundant sugarcane resources to produce bioplastics such as polyethylene (PE) from ethanol derived from sugarcane. Companies such as Braskem have successfully commercialized sugarcane-based PE, which can be recycled through existing mechanical recycling processes, reducing the carbon footprint of plastic production [51,56].

#### 5.2.4. US-Based Compostable Biopolymers in Commercial Food Service

Companies such as EcoProducts and NatureWorks LLC have significantly contributed to the adoption of compostable biopolymers within the food service industry. These companies supply compostable biopolymers to numerous commercial food service sectors, including universities, corporate campuses, healthcare facilities, and large venues such as sports arenas. The motivation for these organizations to purchase and use compostable biopolymers is often driven by zero waste initiatives, plastics bans, and sustainability goals. For instance, in regions where commercial composting infrastructure is available, organizations have successfully diverted a significant portion of their organic waste, including compostable biopolymers, from landfills to composting facilities. This diversion not only reduces the environmental impact but also supports the development of the regional compost market [60].

#### 5.2.5. Starch-Based Bioplastics for Food Preservation

Several projects have shown that starch-based bioplastics work well for preserving food. For example, a notable project developed bioactive films that were composed of banana starch, aloe vera gel, and curcumin. These films greatly improved resistance to water vapor and increased the tensile strength of these films. These bioplastics, designed for wrapping meat products, incorporated antimicrobial properties by integrating essential oils and clay particles, proving effective in extending the shelf life of various food items. In another successful case, cassava starch-based films, combined with rosemary extracts, provided antimicrobial protection and were used for packaging applications. The development of these films showcased the potential of biopolymers to offer both functional benefits and sustainability, addressing the dual need for food safety and environmental responsibility [61].

#### 5.2.6. US-Based Municipal Solid Waste Management and Composting

Several municipalities have also made strides in biopolymer recycling. For instance, the City of Phoenix in the US has implemented a system for managing municipal solid waste, including the composting of biopolymers. Through partnerships with commercial composters and waste management companies, the city has successfully integrated biopolymers into its organic waste diversion programs. This initiative not only supports the local composting industry but also aligns with the city’s sustainability goals of reducing landfill waste and greenhouse gas emissions. Similarly, the state of California’s recent bans on single-use plastics and mandates for compostable products have encouraged the widespread adoption of biopolymers in various sectors. The state’s efforts have been complemented by the development of infrastructure to support the composting of biopolymers, showcasing a comprehensive approach to waste management and sustainability [60].

## 6. Future Directions in Biopolymer Recycling

### 6.1. Innovative Recycling Technologies

The future of biopolymer recycling hinges on the development and implementation of innovative technologies designed to overcome current challenges. One promising area of research is the enhancement of enzymatic degradation processes. Enzymes specific to various biopolymers, such as PETase for polyethylene terephthalate (PET), are being engineered to efficiently break down biopolymers under mild conditions. These enzymes can be tailored to target specific biopolymer structures, reducing contamination and degradation issues commonly faced in mechanical recycling [31,53].

A different noteworthy technology is the development of high-speed precision sorting using artificial intelligence (AI) and computer vision. For instance, AMP Robotics, a smart recycling organisation in the US, has introduced a high-speed robot capable of sorting items at a pace of 160 pieces per minute, significantly improving the efficiency and accuracy of recycling processes. This technology uses AI to recognize and categorize various materials, ensuring that biopolymers are correctly identified and processed [58,62].

Another innovative approach is the use of advanced chemical recycling methods. Solvent-based recycling, which dissolves biopolymers to separate them from contaminants, is gaining attention for its potential to handle mixed waste streams and produce high-purity recycled materials. Additionally, pyrolysis and gasification processes are being explored to convert biopolymer waste into valuable chemicals and fuels, offering a circular economy solution by transforming waste into resources [52].

**Figure 5:**
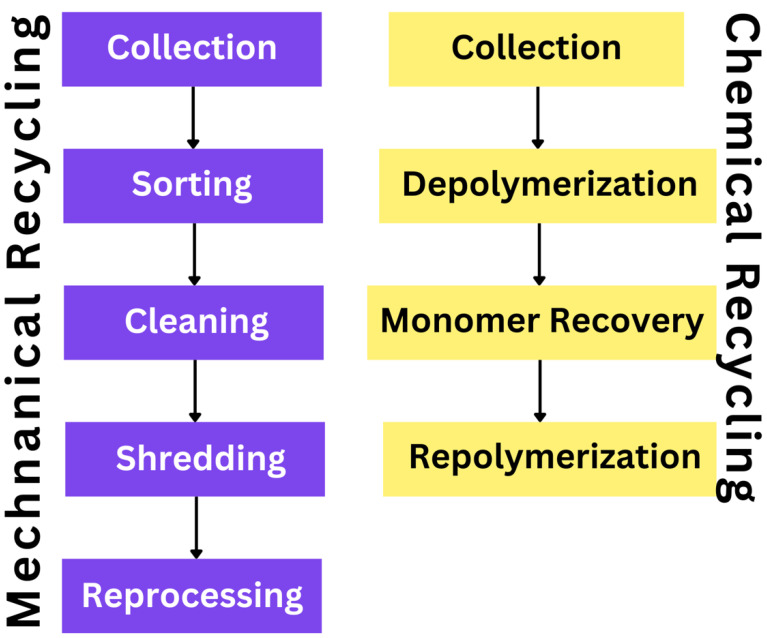
Mechanical and Chemical Recycling Methods for Biopolymers.

Figure 5 illustrates the step-by-step processes involved in both mechanical and chemical recycling methods for biopolymers, highlighting the key stages from collection to reprocessing or repolymerization.

### 6.2. Integration with Circular Economy Models

Integrating biopolymer recycling into broader circular economy models is essential for enhancing sustainability. Circular economy models emphasize the importance of designing products for longevity, reuse, and recyclability. For biopolymers, this means developing materials that are easier to recycle and creating systems that promote their efficient collection and processing.

One approach is the establishment of closed-loop recycling systems, where biopolymer products are designed with end-of-life recycling in mind. This can be facilitated through product design innovations, such as mono-material packaging that simplifies sorting and recycling. Additionally, developing infrastructure for the collection and sorting of biopolymer waste is critical to ensure a steady supply of recyclable materials [63].

Another example of this integration is the concept of agro-waste valorization, where agricultural residues are converted into valuable biopolymers. This approach not only reduces waste but also creates a sustainable source of raw materials for biopolymer production. For instance, researchers have explored the use of citrus processing wastes to produce biopolymers such as polyhydroxyalkanoates (PHAs), which are biodegradable and have numerous industrial applications [64].

Collaboration between stakeholders across the supply chain is also vital. Manufacturers, recyclers, and policymakers must work together to create standardized recycling protocols and incentivize the use of recycled biopolymers. Policies that promote extended producer responsibility (EPR) can encourage manufacturers to design products with recyclability in mind and invest in recycling infrastructure [49].

### 6.3. Collaborations and Partnerships

Advancing biopolymer recycling technologies requires robust collaborations and partnerships among academia, industry, and the government. Research institutions play a crucial role in developing new recycling technologies and providing the scientific basis for their implementation. Industry partnerships are essential for scaling up these technologies and integrating them into existing manufacturing and recycling systems.

For instance, the REMADE Institute in the US, a public-private partnership, has been instrumental in funding research and development projects focused on recycling technologies. One such project involves Braskem, a leading producer of biopolymers, which received a grant to develop new recycling processes for extracting pure polypropylene from PCR materials. This collaboration aims to enhance the recycling infrastructure and promote the use of recycled biopolymers in various industries [65].

Additionally, academic institutions play a vital role in advancing biopolymer recycling research. Collaborative efforts between universities and industry partners have led to significant breakthroughs in biopolymer synthesis and recycling methods. For example, researchers at Aarhus University have developed new NIR (near-infrared) camera technology capable of distinguishing multiple polymer types, which has the potential to revolutionize the recycling industry by improving sorting accuracy [66].

Government support is also critical in creating an enabling environment for biopolymer recycling. Policies that provide financial incentives for recycling initiatives, such as grants, subsidies, and tax breaks, can help overcome economic barriers. Furthermore, regulatory frameworks that promote the use of recycled materials and mandate recycling targets can drive industry adoption of recycling technologies [51].

International collaborations can facilitate the sharing of best practices and technologies across borders. For example, the Ellen MacArthur Foundation’s New Plastics Economy initiative brings together stakeholders from around the world to develop and promote circular economy solutions for plastics, including biopolymers. Such collaborative efforts are essential for accelerating the development and implementation of biopolymer recycling technologies globally [67].

## 7. Conclusions

The future of biopolymer recycling hinges on continuous technological innovation, integration into circular economy models, and stimulating collaborations among key stakeholders in relevant industries. While this review highlights promising recycling technologies such as mechanical, chemical, and enzymatic processes, several limitations must be acknowledged. First, the variability in biopolymer compositions complicates recycling, as different materials often require unique recycling techniques. This variability poses technical challenges in standardizing processes across industries. Additionally, contamination from organic materials, such as food residues, remains a barrier, affecting the quality and usability of recycled biopolymers. Lastly, the economic viability of recycling biopolymers is constrained by high processing costs, limited infrastructure, and market demand for recycled materials. Looking into the future, the focus must shift towards overcoming these limitations by advancing research into high-efficiency recycling technologies and addressing infrastructural and regulatory gaps. Solutions may involve developing materials that are easier to recycle, improving collection and sorting systems, and fostering stronger partnerships between academia, industry, and the government. Through targeted efforts to address these challenges, biopolymer recycling can become a cornerstone of sustainable waste management, significantly reducing plastic pollution and contributing to global environmental goals.

## Figures and Tables

**Table 1:** Types of Polymers.

Source/Method of Production	Types of Biopolymers	Examples	References
Synthesis by microorganisms	Polyesters, Polysaccharides, Polyamides, and Polyphosphates	PHA	[19,20,21]
Derived from animal and plant biomass	Polysaccharides, proteins, and polyesters	Silk, collagen, gelatin, chitosan	[22]
Biotechnology	Polyesters	PLA	[24]

**Table 2:** Examples of Biopolymers and their Applications.

Biopolymer	Properties	Sources	Applications	Industry	
Chitosan	Low molecular weight, biocompatibility, biodegradability, antimicrobial properties	Crab, shrimp, lobster	Wound healing, tissue engineering, food packaging, bone regeneration, and drug delivery	Food, agriculture, and pharmaceutical	[9,26,27]
Cellulose	High mechanical strength, renewability, insolubility in water and various organic solvents	Plant cell walls	Drug delivery and packaging	Pharmaceutical, pulp and paper, and food	[8,26,27]
PHA	Biodegradability, biocompatibility, renewability, high volume-to-surface ratio, good tensile strength, insolubility, and ease of processing	Microbial fermentation of sugar and lipids	Biodegradable packaging, films, laminates, bottles, and containers	Food, agriculture, aerospace, and cosmetics	[19,20,21,32]
Collagen	High molecular weight, biodegradability, and biocompatibility	Animal protein	Wound management, drug delivery, tissue engineering	Pharmaceutical	[13,22,29]
Gelatin	High surface activity, bioavailability, and ease of modification	Animal bones, hides, and skin	Emulsifiers, drug delivery, food stabilization	Food, pharmaceutical	[13,22]
PLA	Biodegradability, high mechanical strength, rigidity, good barrier properties, and light transmission	Microbial fermentation of sugar from plants such as sugar cane, corn, and tapioca	Food packaging, shopping and waste bags, agriculture films, diapers, sanitary towels, and drug delivery systems	Agriculture, food, packaging, and pharmaceutical	[32]
